# Glucuronidated Quercetin Lowers Blood Pressure in Spontaneously Hypertensive Rats via Deconjugation

**DOI:** 10.1371/journal.pone.0032673

**Published:** 2012-03-12

**Authors:** Pilar Galindo, Isabel Rodriguez-Gómez, Susana González-Manzano, Montserrat Dueñas, Rosario Jiménez, Carmen Menéndez, Félix Vargas, Juan Tamargo, Celestino Santos-Buelga, Francisco Pérez-Vizcaíno, Juan Duarte

**Affiliations:** 1 Department of Pharmacology, School of Pharmacy, University of Granada, Granada, Spain; 2 Department of Physiology, School of Medicine, University of Granada, Granada, Spain; 3 Grupo de Investigación en Polifenoles, Facultad de Farmacia, Universidad de Salamanca, Salamanca, Spain; 4 Department of Pharmacology, School of Medicine, Universidad Complutense de Madrid, Instituto de Investigación Sanitaria del Hospital Clínico San Carlos, Madrid, Spain; 5 Ciber Enfermedades Respiratorias, Madrid, Spain; Universidad Federal de Santa Catarina, Brazil

## Abstract

**Background:**

Chronic oral quercetin reduces blood pressure and restores endothelial dysfunction in hypertensive animals. However, quercetin (aglycone) is usually not present in plasma, because it is rapidly metabolized into conjugated, mostly inactive, metabolites. The aim of the study is to analyze whether deconjugation of these metabolites is involved in the blood pressure lowering effect of quercetin.

**Methodology/Principal Findings:**

We have analyzed the effects on blood pressure and vascular function *in vitro* of the conjugated metabolites of quercetin (quercetin-3-glucuronide, Q3GA; isorhamnetin-3-glucuronide, I3GA; and quercetin-3′-sulfate, Q3'S) in spontaneously hypertensive rats (SHR). Q3GA and I3GA (1 mg/kg i.v.), but not Q3'S, progressively reduced mean blood pressure (MBP), measured in conscious SHR. The hypotensive effect of Q3GA was abolished in SHR treated with the specific inhibitor of β-glucuronidase, saccharic acid 1,4-lactone (SAL, 10 mg/ml). In mesenteric arteries, unlike quercetin, Q3GA had no inhibitory effect in the contractile response to phenylephrine after 30 min of incubation. However, after 1 hour of incubation Q3GA strongly reduced this contractile response and this effect was prevented by SAL. Oral administration of quercetin (10 mg/Kg) induced a progressive decrease in MBP, which was also suppressed by SAL.

**Conclusions:**

Conjugated metabolites are involved in the *in vivo* antihypertensive effect of quercetin, acting as molecules for the plasmatic transport of quercetin to the target tissues. Quercetin released from its glucuronidated metabolites could be responsible for its vasorelaxant and hypotensive effect.

## Introduction

Flavonoids are polyphenolic compounds that occur ubiquitously in plants and are consumed in the form of fruits, vegetables, nuts and derived products such as wine and chocolate. The average daily intake in the western diet of flavonols plus flavones (two main classes of flavonoids) is estimated to be ≈23 mg, with quercetin (3,3′,4′,5,7-pentahydroxyflavone) contributing 60–75% of the total [1], [2]. Quercetin is a prime example of such a flavonoid group and it is found in foods bound to sugars, mainly as β-glycosides. Quercetin glycosides occur in broccoli, apples, and especially in onions, with an abundance as high as 0.25–0.5 g/kg [3]. Prospective studies have shown an inverse correlation between dietary flavonoid intake and mortality from coronary heart disease [1], [4]. Several studies using various animal models provide support for the observed protective effects of dietary flavonoids with respect to cardiovascular diseases [5]. For example, quercetin exerts systemic and coronary vasodilatation and antiaggregant effects *in vitro* [6]–[8] and reduces blood pressure, oxidative status and end-organ damage in animal models of hypertension [9], [10], including spontaneously hypertensive rats (SHR) [9]–[12]. Chronic quercetin also reduces blood pressure in stage 1 hypertensive subjects [13]. However, there are not studies analyzing the acute effects on blood pressure of oral quercetin.

**Figure 1:**
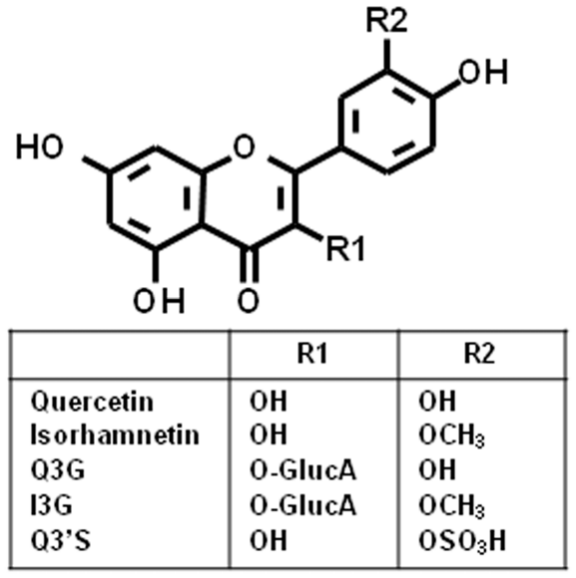
Structure of quercetin and its metabolites isorhamnetin, quercetin 3-glucuronide (Q3GA), isorhamnetin 3-glucuronide (I3GA) and quercetin 3′-sulfate (Q3'S).

Many previous *in vitro* studies have exposed tissues or cultured cells to commercially available aglycones or the glycosylated compounds which are present at extremely low concentrations in plasma [14]. Upon ingestion with the diet, quercetin glycosides are rapidly hydrolyzed during their passage across the small intestine or by bacterial activity in the colon to generate quercetin aglycone. Absorbed quercetin is rapidly conjugated with glucuronic acid and/or sulfate during first-pass metabolism (intestine-liver) and a portion of the metabolites are also methylated and, therefore, the major metabolites of quercetin in rat and human plasma are quercetin-3-glucuronide (Q3GA), quercetin-3′-sulfate (Q3'S) and isorhamnetin-3-glucuronide (I3GA) (Figure 1) while the aglycone is usually undetectable [15]–[18]. The biological activity of quercetin is generally attenuated after its conversion into the metabolites. However, antioxidant activity for various quercetin metabolites has been reported [19]–[21]. This may lead in vascular beds to an improvement of endothelial function, while the conjugated metabolites have no direct vasorelaxant effect in rat aorta [21]. Moreover, injured/inflamed arteries, as occur in hypertension and atherosclerosis, with activated macrophages are potential targets of the metabolites of dietary quercetin [22]. Some previous studies have shown that quercetin glucuronides can be deconjugated *in vitro* in cultured macrophages [22] and in homogenates from human liver and small intestine [23]. Q3GA can be also slowly deconjugated within the vascular wall [24].

We hypothesized that the antihypertensive effects of quercetin could be mediated by the conjugated derivatives that are present in the circulating blood. These metabolites would reduce vascular tone after deconjugation in the vascular tissue. Therefore, the aim of the present study was to analyse the long term *in vitro* effects of the main plasma quercetin conjugates in resistance mesenteric arteries, their *in vivo* effects given intravenously on blood pressure in SHR and the role of deconjugation via glucuronidase. Moreover, we tested whether deconjugation is required for the antihypertensive effects of oral quercetin aglycone.

## Materials and Methods

### Animals

All the experiments were performed in accordance with Institutional Guidelines for the ethical care of animals, and ethic committee of the University of Granada approved this study (ref. 2066/10). Twenty four-week old, male spontaneously hypertensive rats (SHR) were obtained from Harlan Laboratories (Barcelona, Spain). All rats were maintained five per cage at a constant temperature (24±1°C), with a 12-hour dark/light cycle and on standard rat chow.

### Blood pressure measurement

Direct blood pressure was measured in conscious SHR. For this purpose, the rats were anaesthetised with 2.5 mL/kg i.p. equitensin (500 mL contain 43% w/v chloral hydrate in 81 mL ethanol; 4.86 mg pentobarbitone; 198 mL propylene glycol; 10.63 g MgSO_4_; distilled water) and the carotid artery was cannulated to obtain direct measurements of arterial blood pressure. The catheter was exteriorised through the skin on the dorsal side of the neck and protected with a silver spring. A cannula was also introduced into the left jugular vein for the administration of quercetin metabolites and blood sampling. Upon completion of the surgical procedure, rats were fasted and allowed to recover for 6 h and, after connecting the catheter to a transducer and a two-channel recorder (TRA-021 and Letigraph 2000, respectively; Letica SA, Barcelona, Spain), blood pressure and heart rate (HR) were continuously recorded. Animals received either Q3GA (0.2, 0.02 or 1 mg/kg), Q3'S or I3GA (1 mg/kg), or drug vehicle (100 µL of phosphate buffered saline). The acute effect of an oral dose of quercetin (10 mg/kg) administered by gavage on blood pressure and heart rate were also analysed.

In another set of experiments, SHR rats were daily given i.p. for 3 days either isotonic solution (1 mL) or D-saccharic acid 1.4-lactone (SAL), a specific inhibitor of beta-glucuronidase, (10 mg/mL in 1 mL) [25] before the administration of the flavonoids.

### Analysis of quercetin metabolites in rat plasma

Blood was collected into heparinized tubes and centrifuged. The plasma samples (300 µL) were extracted with 300 µL of methanol/0.5 M acetic acid (80∶20, v/v) for 30 min at 25°C in an ultrasonic bath, and then centrifuged for 3 min at 3500 g. The supernatant was collected and the pellet was submitted to the same process two further times assisted by sonication (1 min) using a MicrosonTM ultrasonic cell disruptor (New York, USA). The methanolic extracts were combined and dried in a centrifugal concentrator micVac (GeneVac, Ipswich, United Kingdom). The residue was dissolved in 120 µL acetonitrile/water (30∶70 v/v) and centrifuged (5 min, 3500 *g*) previous to its injection (100 µL) in the HPLC-DAD-ESI/MS system.

Analyses were carried out in a Hewlett-Packard 1100 chromatograph (Agilent Technologies, Waldbronn, Germany) with a quaternary pump and a DAD coupled to an HP Chem Station (rev. A.05.04) data-processing station. An Ascentis™ RP-Amide 3 µm (2.1×150 mm) column at 30°C was used. The solvents used were: (A) 0.1% formic acid, and (B) acetonitrile. An elution gradient was established from 15 to 50% B over 15 min, isocratic 50% B for 10 min, from 50 to 75% B over 3 min, isocratic 75% B for 10 min, and re-equilibration of the column, at a flow rate of 0.2 mL/min. Double online detection was carried out in the DAD using 370 nm as a preferred wavelength and in a mass spectrometer connected to HPLC system via the DAD cell outlet. MS detection was performed in an API 3200 Qtrap (Applied Biosystems, Darmstadt, Germany) equipped with an ESI source and a triple quadrupole-ion trap mass analyzer that was controlled by the Analyst 5.1 software. Zero grade air served as the nebulizer gas (30 psi) and turbo gas for solvent drying (400°C, 40 psi). Nitrogen served as the curtain (20 psi) and collision gas (medium). The quadrupoles were set at unit resolution. The ion spray voltage was set at −4500 V in the negative mode. Precursor ion analysis was employed to detect all the precursor ions that fragment to a common product ion (i.e., m/z 301 corresponding to quercetin). Settings used were: declustering potential (DP) −40 V, entrance potential (EP) −10 V, collision energy (CE) −50 V, and cell exit potential −3 V. Enhanced product ion mode was further performed in order to obtain the fragmentation pattern of the parent ion(s) of the studied transition in the previous experiment using the following parameters: DP −50 V, EP −6 V, CE −25 V, and collision energy spread 0 V. Quantitative analysis of the assayed flavonols and conjugated metabolites was performed from their chromatographic peaks recorded at 370 nm by comparison with calibration curves obtained by injection of increasing concentrations of quercetin, I3GA, and Q3GA.

### Vascular reactivity *in vitro*

SHR were stunned and killed by cervical dislocation. The mesentery was removed and placed in cold Krebs solution (composition in mmol/L: NaCl 118, KCl 4.75, NaHCO_3_ 25, MgSO_4_ 1.2, CaCl_2_ 2, KH_2_PO_4_ 1.2, and glucose 11). Third-order arteries were cleaned of surrounding fat and mounted in an automated tension myograph (Danish Myotechnology, Denmark) containing Krebs solution maintained at 37°C and gassed with 5% CO_2_ in O_2_. After an equilibration period of 45 min, vessels were normalized according to published protocols and vessel diameter determined [26]. Following normalization, relaxation of phenylephrine (3 µM)-precontracted vessels to acetylcholine (Ach, 1 µM) was used to determine endothelial integrity (vessels that relaxed by at least 50% were considered endothelium-intact).

In order to analyze the effects on vascular function, in endothelium-intact rings a concentration–response curve was constructed by cumulative addition of phenylephrine (10^−7^–10^−4^ M). Then vessels incubated in the absence or presence of quercetin, isorhamnetin, Q3'S, Q3GA or I3GA (10 or 25 µM) for 30–120 min and a second concentration–response curve was performed. In some arteries SAL (1 mM) was added 1 hour before and during the incubation period with Q3GA.

### β-glucuronidase activity

β-glucuronidase activity was measured by a colorimetric analysis using phenolphthalein mono-β-glucuronide as the substrate [22]. Briefly, 30 µg of protein of vascular mesenteric bed homogenates from SHR were mixed with 0.6 mM phenolphthalein mono-β-glucuronide in 100 µL of 0.1 mM sodium phosphate buffer at pH 5. After incubation at 37°C for 30 min followed by adding 200 µL of 0.1 M sodium phosphate buffer pH 11, the absorbance at 540 nm indicating the formation of phenolphthalein aglycone was measured. In some experiments, SAL (1 mM) was added 1 hour before phenolphthalein mono-β-glucuronide addition.

### Materials

Q3GA was isolated from green bean pods and stored as described [27]. Briefly, defated pods were homogenized in 70% MeOH, the concentrated extract was fractionated on a polyamide column and washed firstly with phosphate buffer, then with methanol and finally with methanol/ammonia (99.5∶0.5 v/v) to elute the acidic flavonols (e.g. glucuronides). The glucuronide was purified by semipreparative-HPLC. Q3'S was synthesized by an adaptation of the method described by Jones et al. [28]. Briefly, dehydrated quercetin was dissolved in dioxane and allowed to react at 40°C for 90 min with a 10-fold molar excess of sulfur trioxide-N-triethylamine complex under a nitrogen atmosphere. Precipitated products of sulfation were redissolved in 10% methanol in water and the mixtures of quercetin sulfates were fractioned on a Sephadex LH-20 column and Q3'S further purified by semipreparative HPLC. I3GA was produced enzymatically using pig liver microsomal enzymes with a modification of the methodology described by Plumb et al. [29]. Briefly, a post-lysosomal fraction was obtained from a pig liver extract, incubated with isorhamnetin at 37°C for 240 min, in a Hepes buffer (25 mM, pH 5.5 and pH 7.2) or ultra-pure water, containing in either case 10 mM MgCl_2_, UDP-glucuronic acid (8 mM) and UDP-glucosamine (4 mM). I3G was isolated by semipreparative HPLC. All other drugs were from Sigma (Tres Cantos, Madrid, Spain).

### Statistical analysis

Results are expressed as the mean ± SEM and n describes the number of measurements made (i.e., from different animals). Differences between experimental groups were treated using unpaired Student's t-test or, for multiple comparisons, using one-way analysis of variance followed by a Dunnett's post hoc test. P values<0.05 were considered statistically significant.

## Results

### Effects of plasma metabolites on blood pressure

**Figure 2:**
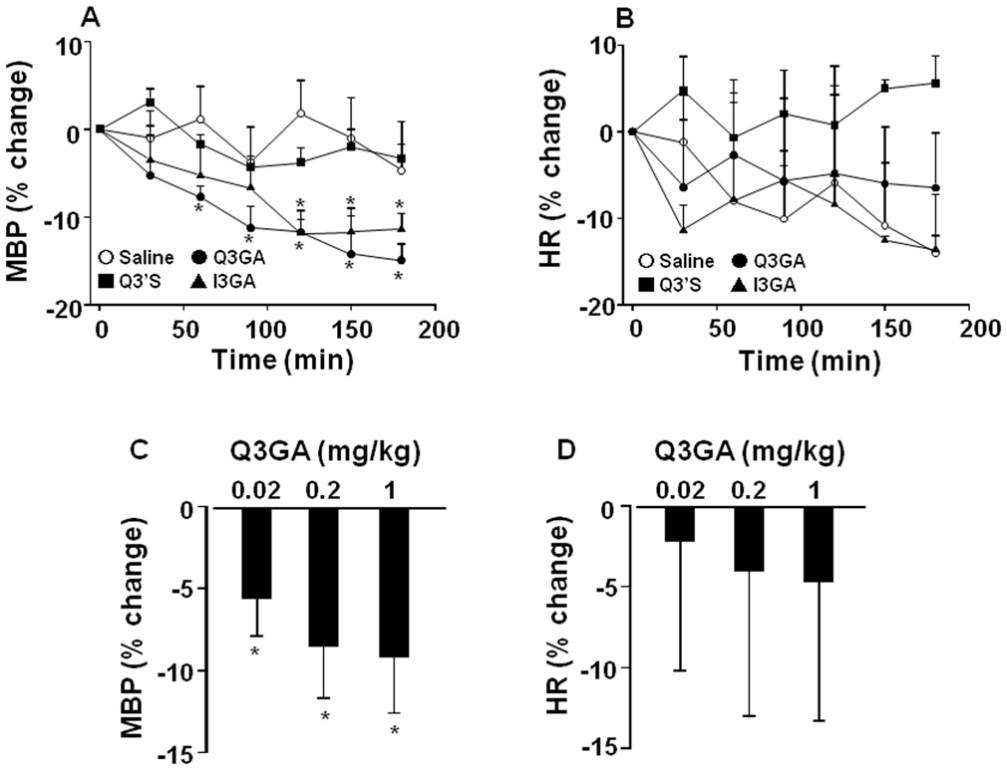
Effects of intravenous Q3GA, Q3'S, and I3GA (1 mg/kg) and Q3GA (0.02, 0.2 mg/kg) on mean arterial blood pressure (A, C) and heart rate (B, D) measured by direct carotid artery recording in a conscious rat. Results are means ± SEM of 4–6 experiments. * P<0.05 vs. saline.

SHR showed a basal MBP of 181±5 mm Hg and HR of 424±14 bpm. Q3GA and I3GA (1 mg/kg i.v.) progressively reduced mean blood pressure (MBP) in SHR, while Q3'S was without effect. This hypotensive effect induced by both metabolites was statistically significant after 1 and 2 h, respectively, of the metabolite injection. The maximum effects observed at 3 h were 14.9±1.8% and 11.4±1.8%, respectively (Figure 2A). No significant changes in heart rate (HR) were observed with any metabolite (Figure 2B). Q3GA also decreases MBP at low concentrations (0.02 and 0.2 mg/kg) (Figure 2C), being also without effects on HR (Figure 1D).

### Time-course of the Q3GA concentrations in plasma

**Figure 3:**
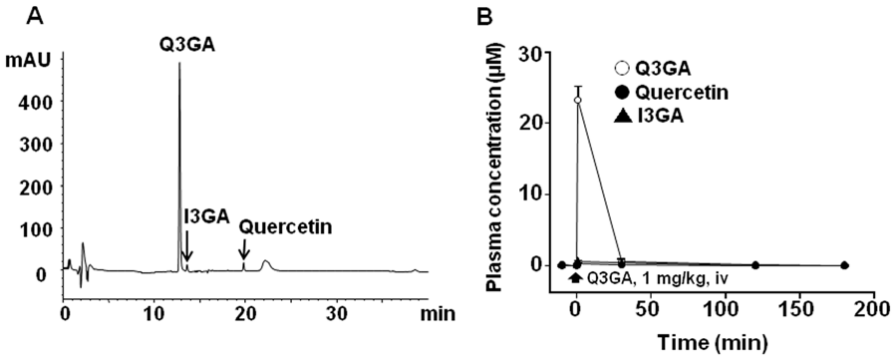
Concentrations of quercetin, I3GA, and Q3GA measured in plasma from SHR treated with 1 mg/kg Q3GA. (A) HPLC chromatograms recorded at 370 nm of plasma samples taken at 1 min. (B) Time-concentration relationship. Results are means ± SEM of 4 experiments.

When SHR were treated with Q3GA, 1 mg/kg i.v., there was an increase in the plasma concentration of this metabolite reaching 23.2±1.8 µM at 1 min and decreased rapidly (<1 µM at 30 min) (Figure 3A, 3B). Moreover, free quercetin aglycone and I3GA was detected in plasma after Q3GA injection.

### Effects of metabolites in the reactivity of mesenteric artery

**Figure 4:**
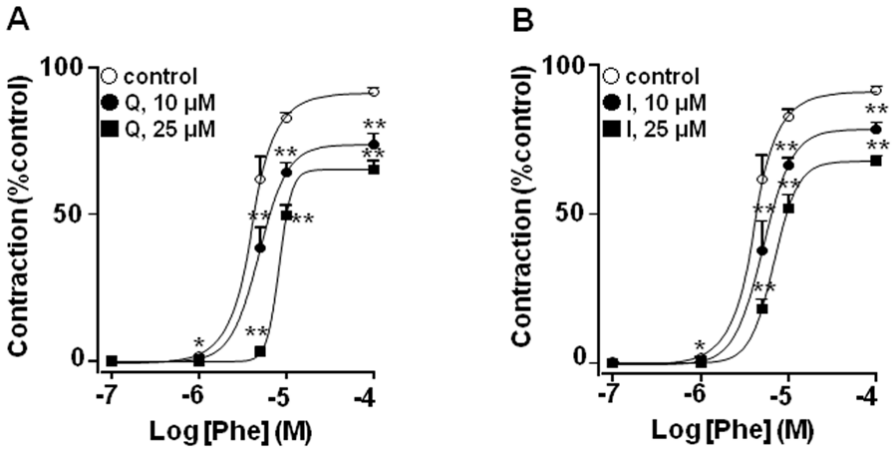
Effects of (A) quercetin and (B) isorhamnetin (10 or 25 µM, incubated for 30 min) on the contractile responses to phenylephrine in mesenteric resistance arteries. Control is treated with vehicle (DMSO). Results are means ± SEM of 4–8 experiments. * P<0.05 and **P<0.01 vs. control.

**Figure 5:**
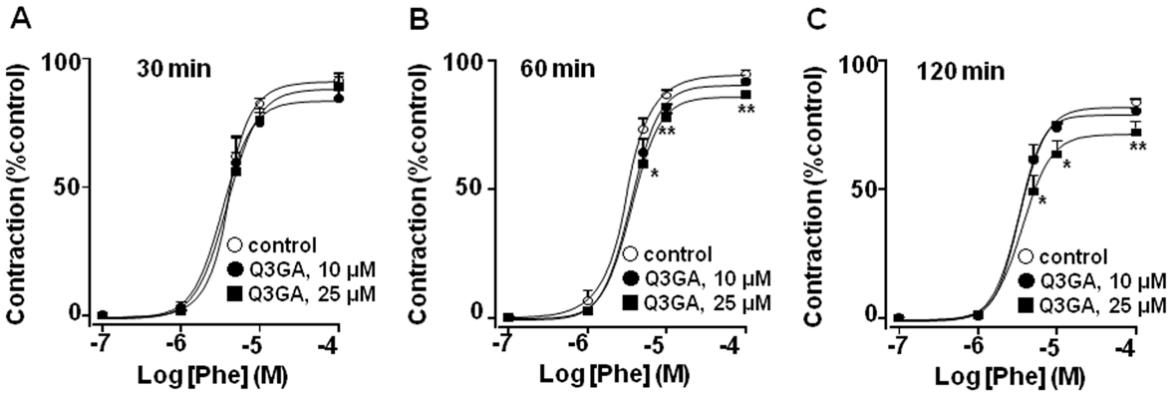
Effects of Q3GA (10 or 25 µM) on the contractile responses to phenylephrine in mesenteric resistance arteries, after 30 (A), 60 (B) or 120 (C) min of incubation. Results are means ± SEM of 4–8 experiments. * P<0.05 and **P<0.01 vs. control.

Phenylephrine induced a maximal contractile effect in mesenteric vessels from SHR of 19.4±0.9 mN (n = 20). When mesenteric arteries from SHR were incubated with the aglycones quercetin or isorhamnetin for 30 min a significant concentration-dependent decrease in the vasoconstrictor response to phenylephrine was observed (Figure 4) while Q3GA at this time had no effect (Figure 5A). However, when the incubation of 25 µM Q3GA was prolonged to 1 and 2 hours a significant reduction in the vasoconstriction induced by phenylephrine was detected (Figure 5B and 5C).

### Role of glucuronidase activity in the hypotensive and vascular effects induced by Q3GA

**Figure 6:**
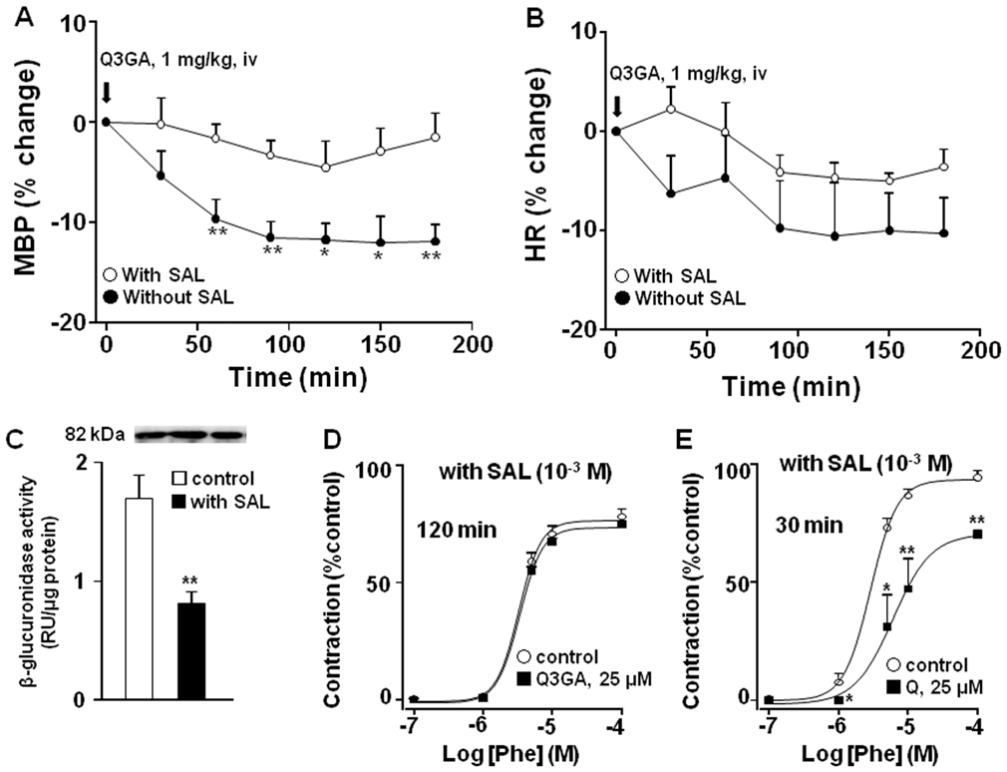
Effects of Q3GA in arterial blood pressure (A) and heart rate (B) in SHR treated with SAL (10 mg/rat/day for 3 days) or vehicle (means ± SEM of 4 experiments). Panel (C) shows the bands of β-glucuronidase expression by Western blot and the β-glucuronidase activity and its inhibition by SAL (1 mM) in vascular bed homogenates (means ± SEM of 8 experiments). (D) Effects of Q3GA (25 µM) on the contractile responses to phenylephrine in mesenteric arteries after 120 min in the presence of SAL (1 mM) (means ± SEM of 5 experiments). (E) Effects of quercetin (25 µM) on the contractile responses to phenylephrine in mesenteric arteries after 30 min in the presence of SAL (1 mM) (means ± SEM of 5 experiments). * P<0.05 and **P<0.01 vs. control.

To explore the possible role of deconjugation of Q3GA on the observed effects a specific inhibitor of beta-glucuronidase (SAL) was used, which was administered i.p. during the 3 days before the blood pressure recordings. Interestingly, the hypotensive effect of Q3GA was abolished in SHR treated with SAL (Figure 6A). We confirmed the inhibitory glucuronidase activity of SAL (1 mM) in homogenates from the mesenteric bed in *in vitro* conditions, by incubating during 1 h and measuring glucuronidase activity (Figure 6C). We also found that the inhibitory effects of Q3GA in the contractile response induced by phenylephrine were suppressed when mesenteric arteries were incubated with SAL (Figure 6D), but not those of quercetin (Figure 6E).

### Role of glucuronidase activity in the hypotensive and vascular effects induced by quercetin

**Figure 7:**
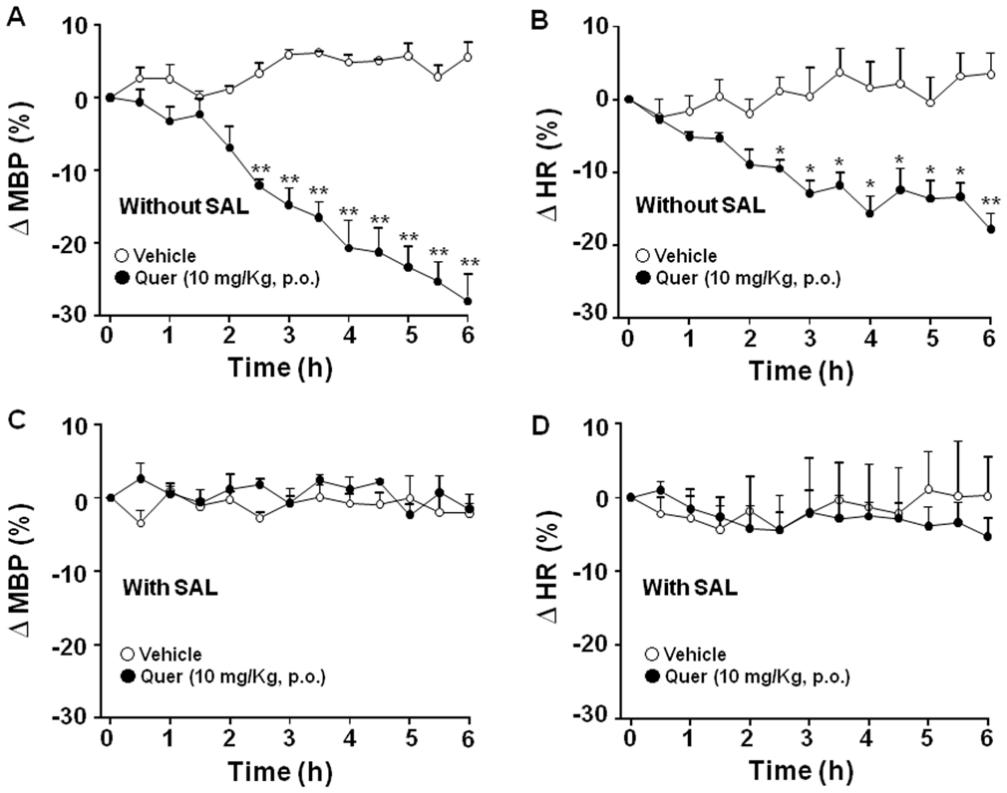
Effects of oral quercetin (10 mg/kg) on arterial blood pressure (A, C) and heart rate (B, D) in SHR treated with SAL (10 mg/rat/day for 3 days) or vehicle (1 ml isotonic solution). Results are means ± SEM of 4 experiments. *P<0.05 and **P<0.01 vs. quercetin vehicle (1 ml of 1% methylcellulose).

The above results prompted us to analyze whether deconjugation was also required for the antihypertensive effect of orally administered quercetin. Administration of quercetin (10 mg/Kg using an intragastric gavage) induced a progressive decrease in MBP and HR during 6 hours of register. These reductions were significant, as compared to vehicle, after 2.5 h of administration, and reached a maximum of 28±4% and 18±2%, respectively at 6 h (Figure 7A, 7B). Importantly, when SHR were treated with SAL, oral quercetin was unable to induce changes in both MBP and HR (Figure 7C, 7D). However, SAL was unable to modify the in vitro effect of quercetin on the contractile response to phenylephrine in isolated mesenteric arteries.

## Discussion

Fruit and vegetable consumption is associated with a decrease in blood pressure, which is an important cardiovascular risk factor [30]. Quercetin, the most important dietary flavonol, present in multiple fruits and vegetables, reduces blood pressure in hypertensive animals and human after chronic consumption [9], [13], [31]–[37]. Herein, we show for the first time that the conjugated derivatives Q3GA and I3GA can exert antihypertensive effects when administered intravenously. As previously reported Q3GA had no acute effect *in vitro* (at 30 min), however it developed with more prolonged incubations. Both the *in vitro* and the *in vivo* effects were prevented by the β-glucuronidase inhibitor SAL. Taken together these data strongly suggest that deconjugation is required for the effect of quercetin metabolites. Moreover, oral quercetin reduced blood pressure by almost 30% in SHR, being this effect persistent at least during 6 hours and, importantly, this effect was also prevented by SAL, indicating that the sequence of liver-intestine conjugation and local (vascular) deconjugation processes is required for the antihypertensive effect of quercetin.

Both human and rat tissues, except for the cells lining the intestine tract, are exposed to quercetin via the blood. However, the free forms of quercetin and its methylated metabolite isorhamnetin are barely detected in plasma, which raises the question of which is/are the compound(s) responsible for the antihypertensive activity. Because glucuronidated and sulfated compounds are the only detectable metabolites, it is suggested that conjugated metabolites must play a decisive role in the possible beneficial effects [38]. Our results support this hypothesis, because we showed that Q3GA and I3GA, the main plasma metabolites of quercetin exerted an antihypertensive effect. Doses of Q3GA as low as 0.02 mg/kg also significantly reduced blood pressure. In contrast, Q3'S was without effect. Herein, we show that both Q3GA and I3GA metabolites show a similar effect. Previous published papers from other groups [15]–[18], the concentrations of methylated forms of quercetin are in the same range or higher than non methylated ones in rats and humans supplemented with quercetin, suggesting that both forms may contribute to the antihypertensive effect.

When we analyzed the time course of the antihypertensive effect and compared it to the plasma concentrations of Q3GA we found a clear dissociation (Fig. 2A vs Fig. 3B). In our experimental conditions, the dose of 1 mg/kg of Q3GA intravenously induced a peak plasma concentration of ∼25 µM which is higher than that previously reported by da Silva et al. [39] of 9.6 µM 6 h after 10 mg/kg quercetin delivered via oral gavage. However, Q3GA rapidly disappeared from the plasma, indicating that the two modes of administration result in a completely different pharmacokinetic profile. The fast decay of Q3GA concentration in plasma is not compatible with renal excretion, suggesting that Q3GA is metabolized, accumulated in tissues or both. In a recent *in vitro* study [24], the perfusion of Q3GA through the rat mesenteric vascular bed resulted in a partial accumulation of Q3GA in the tissue and a progressive process of deconjugation. The resulting aglycone was partly found in the extracellular buffer and mostly retained intracellularly. The beta-glucuronidase inhibitor SAL increased the tissue Q3GA and reduced the aglycone. Deconjugation by beta-glucuronidase is expected to occur intracellularly because this enzyme is located in the lysosomes and the microsomal fraction. Therefore, the aglycone is formed within the vessel and probably in the cytosol of smooth muscle cells where it is expected to interact with its targets to exert vascular smooth muscle relaxation. The most plausible targets for this effect include the protein kinases involved in the regulation of myosin-actin interactions including protein kinase C, myosin light chain kinase or Rho kinase and possibly potassium channels [8], [9], [40], [41]. Quercetin aglycone released to the plasma is likely to be rapidly re-conjugated in the liver explaining its low levels.

The vasorelaxant effects of quercetin and related metabolites have been widely assessed *in vitro* in aorta and perfused mesenteric bed [7], [8], [42]. Increased alpha-adrenergic response in small mesenteric arteries has been involved in increased blood pressure in SHR [43], [44]. As expected, both quercetin and isorhamnetin incubated during 30 min, inhibited the contractile response induced by the alpha-adrenergic receptor agonist phenylephrine. In the same experimental conditions, Q3GA did not modify this response. These results are consistent with previous data showing that conjugation of flavonoids results in a decreased biological activity [45], [46] and that conjugated metabolites have no direct vasorelaxant effect in isolated rat aorta at physiological concentrations [21]. However, when small mesenteric arteries were incubated for 1 or 2 h with Q3GA, at 25 µM, the vasoconstriction induced by phenylephrine was significantly reduced, suggesting that quercetin accumulates in this vascular bed and it is responsible of the reduced vascular tone. A similar scenario has been described previously in which quercetin metabolites in circulating blood can permeate through the injured/activated endothelial cells and interact with the subintimal cells, such as the macrophages and smooth muscle cells [22], [47]. Deconjugation of the glucuronide metabolites of the flavonoids by increased β-glucuronidase activity at the site of inflammation has been suggested as a plausible mechanism for the protective effects of flavonoids *in vivo* [23], [48]. Accordingly, the release of β-glucuronidase is considered an index of lysosomal membrane integrity [49]. In fact, mesenteric bed from SHR expresses β-glucuronidase and its activity was significantly inhibited by SAL, a specific inhibitor. Vascular tissues from SHR showed increased expression of proinflammatory markers, altered endothelial function, and increased macrophage infiltration than normotensive animals [50], [51], which could facilitate metabolite accumulation and deconjugation in this inflamed tissue. In our experiments, the antihypertensive effect of Q3GA was abolished by β-glucuronidase inhibition, which suggests that this effect requires β-glucuronidase-mediated deconjugation. Moreover, the inhibitory effect in the contractile response to phenylephrine in mesenteric arteries induced by Q3GA was also suppressed by SAL, showing that Q3GA requires deconjugation to exert this inhibitory effect.

Given the role of β-glucuronidase in the effects of Q3GA we aimed to analyze whether it was also relevant for the antihypertensive effect of quercetin. Surprisingly, despite several chronic studies, to our knowledge the effects of acute oral quercetin administration on blood pressure in hypertensive animals had not been studied. We found a slow developing but long lasting antihypertensive effect. Remarkably, the effects of oral quercetin were also abolished by β-glucuronidase inhibition with SAL. However, as expected, the *in vitro* effects of quercetin were unaffected by SAL. Thus, our data suggest that the biological activity of quercetin is dependent on the conjugation-deconjugation processes. Although decreased glucuronidation often results in increased activity and/or toxicity of drugs, paradoxically, glucuronidation seems to be required for the activity of quercetin. Therefore, glucuronidation may protect quercetin from its metabolism via other pathways and help to carry the flavonoid to the tissues where the free aglycone is released [52]. Our data also suggest that polymorphisms of UDP-glucuronosyltransferases (encoded by the UGT1 and UGT2 loci), which are common in humans [53] and changes in the β-glucuronidase activity, might result in a variable response to quercetin.

In conclusion, we show that glucuronides of quercetin and its methylated metabolite isorhamnetin are involved in the antihypertensive response of oral quercetin, which might be related, at least in part, by the inhibitory effect in the α-adrenergic-induced hypercontractile response in resistance arteries. Quercetin could be initially inactivated by a conjugation metabolism during absorption and then safely be delivered to inflamed arterial wall, and the recruited metabolites are incorporated and converted to the aglycone in vascular smooth muscle cells and exert the inhibitory activity on vascular tone. These results are in agreement with the hypothesis that flavonoid glucuronides appear to serve as plasma transport metabolites to target cells rather than solely as excretion.
